# In silico ranking of phenolics for therapeutic effectiveness on cancer stem cells

**DOI:** 10.1186/s12859-020-03849-z

**Published:** 2020-12-28

**Authors:** Monalisa Mandal, Sanjeeb Kumar Sahoo, Priyadarsan Patra, Saurav Mallik, Zhongming Zhao

**Affiliations:** 1 Department of School of Computer Science and Engineering, Xavier University, Bhubaneswar, Odisha 752050 India; 2 Institute of Life Sciences, Bhubaneswar, Odisha 751023 India; 3 Center for Precision Health, School of Biomedical Informatics, The University of Texas Health Science Center At Houston, Houston, TX 77030 USA; 4 Human Genetics Center, School of Public Health, The University of Texas Health Science Center At Houston, Houston, TX 77030 USA

**Keywords:** Phenolics, Cancer stem cell, Bipartite graph, Page rank

## Abstract

**Background:**

Cancer stem cells (CSCs) have features such as the ability to self-renew, differentiate into defined progenies and initiate the tumor growth. Treatments of cancer include drugs, chemotherapy and radiotherapy or a combination. However, treatment of cancer by various therapeutic strategies often fail. One possible reason is that the nature of CSCs, which has stem-like properties, make it more dynamic and complex and may cause the therapeutic resistance. Another limitation is the side effects associated with the treatment of chemotherapy or radiotherapy. To explore better or alternative treatment options the current study aims to investigate the natural drug-like molecules that can be used as CSC-targeted therapy. Among various natural products, anticancer potential of phenolics is well established. We collected the 21 phytochemicals from phenolic group and their interacting CSC genes from the publicly available databases. Then a bipartite graph is constructed from the collected CSC genes along with their interacting phytochemicals from phenolic group as other. The bipartite graph is then transformed into weighted bipartite graph by considering the interaction strength between the phenolics and the CSC genes. The CSC genes are also weighted by two scores, namely, DSI (Disease Specificity Index) and DPI (Disease Pleiotropy Index). For each gene, its DSI score reflects the specific relationship with the disease and DPI score reflects the association with multiple diseases. Finally, a ranking technique is developed based on PageRank (PR) algorithm for ranking the phenolics.

**Results:**

We collected 21 phytochemicals from phenolic group and 1118 CSC genes. The top ranked phenolics were evaluated by their molecular and pharmacokinetics properties and disease association networks. We selected top five ranked phenolics (Resveratrol, Curcumin, Quercetin, Epigallocatechin Gallate, and Genistein) for further examination of their oral bioavailability through molecular properties, drug likeness through pharmacokinetic properties, and associated network with CSC genes.

**Conclusion:**

Our PR ranking based approach is useful to rank the phenolics that are associated with CSC genes. Our results suggested some phenolics are potential molecules for CSC-related cancer treatment.

## Background

Cancers diagnosed at the earlier stage can be curable through conventional treatments such as surgery, chemotherapy and radiotherapy [1–4]. However, cancers diagnosed at a later stage are more progressive and aggressive and they often lead to metastasis to multiple organs. While significant progress has been made to improve diagnosis and surveillance, this has not helped much to improve the overall cancer survival rates [5, 6]. Even after the cancer is diagnosed and treated at earlier stage, not all cancer cells can be killed and tumor recurrence has been frequently reported. When tumor recurrence happens, cancer becomes more aggressive and metastatic [7–9]. Growing evidences [10–12] has indicated that these residual cells play a crucial role as therapeutic resistant and own the property of self-renewal (stem-like properties) known as the cancer stem cells (CSCs). CSCs behave same as normal stem cells do. Moreover, they have multi-differentiative potentials and capa-bility of generating multiple cancer cell types that eventually develop tumors. The self-renewal property of CSCs enables them to give rise to other type malignant cells [13, 14]; therefore, they can be described as phenotypically and functionally diversified immortal tumor cells. Such cells have been found in various types of human tumors and might be attractive targets for cancer treatment [11, 12, 15–17]. These CSCs generally make up just 1% to 5% of all cells in a tumor [18]. Most CSCs are believed to be resistant to chemo- or radio-therapy, indicating CSCs play an important role in cancer relapse and metastasis. Therefore, it requires the development of novel, diverse, and multi-targeted approaches for cancer treatment due to the fact that CSCs have different and still uncovered characteristics. But in fact, clinicians are still struggling to find such CSC targeting therapies with no or limited side-effects.

The currently available treatment options for cancer are surgery, radiation therapy and chemotherapy. More recently, systemic chemotherapy [2, 19–21] has becoming the popular one for cancer treatment. Along with cancer cells, healthy cells are also damaged by chemotherapeutic drugs. This may cause side effects to the patients. Lack of major progresses in molecular targeted therapies has made researchers to unfold the prospects of natural anticancer agents from plants known as phytochem-ical. During the years, phytochemicals are a major topic of research because of their naturally healing capability. For the disease such as cancer, they have been testified for having the potential to target heterogeneous populations of cancer cells and CSCs. Moreover, they are capable of targeting the key signaling pathways of can-cer leaving the normal cells intact or minimal toxicity. However, laboratory-based experiments for identifying the drug targets for natural products is not only ex-pensive, labor expensive, but also a prolonged process. Therefore, computational approaches for drug (phytochemical) ranking can greatly speed up the traditional drug discovery process [15, 22], and can provide potential candidates for follow up experimental validation. To date, there have been strong needs to develop a sys-tematic and comprehensive computation-based approaches to identify and validate phytochemical for cancer cells.

In this study, CSC genes and their interacting phytochemicals from the phenolic group are systematically collected and curated from the available databases. Then, a bipartite graph has been built from the collected data where CSC genes form one disjoint independent set and the interacting phytochemical is the other set. The graph is then weighted according to the interaction strength between the phenolics and the CSC genes. Two different metrics have been used to weight the CSC genes: *DSI*, which indicates the extent of a gene being specific to a disease, and *DP I* which indicates the association of a gene with a set of diseases (pleiotropy). After forming the weighted bipartite graph, a ranking technique based on PageRank (PR) has been applied to rank the phenolics signifying their influence on the CSC genes. Different datasets and platforms are used to validate the resultant phenolics.

## Methods

CSCs, like all stem cells, are unspecialized and can divide and renew themselves, as well as give rise to specialized cells. This type of stem cells can be found in a small proportion within a tumor and can replicate tumor cells. Thus, they may lead to tumor growth and migration. They can be left behind even after the course of cancer treatment completes, allowing the tumor to recur and spread around the body. Natural products may be the one reliable option to discover novel treatments demanded by the difficulty of treating CSCs. The work on CSCs is still in early stages. Currently, the research on CSCs is primarily taking place in the research laboratory. Early clinical trials are targeted in the development of effective anti-cancer strategies. As the number of the experiments is few; therefore, the CSCs related databases [23] are also rare. Moreover, those databases have little CSC related information.

### CSC related genes data

We collected 1118 CSC related genes from the CSCdb database https://bioinformatics.ustc.edu.cn/cscdb [23]. CSCdb is a literature-based database (collected from about 13,000 articles) and useful for CSC-related research. The database contains CSCs marker genes, CSCs-related genes and their functional annotations. It could be an important resource for finding new CSCs and their potential therapeutic tar-gets. A complete information of 1769 genes that have been found to be associated in the functional regulation of CSCs is provided by CSCdb. In addition, 74 marker genes along with 9475 annotations on 13 CSC-related functions have been reported.

### Phenolics data

In addition to the common cancer treatments (surgery, radiotherapy and chemother-apy), the systemic chemotherapy has become an alternative cancer treatment. Two common problems associated with chemotherapy are drug resistance and toxicity by damaging healthy cells, causing them to secret proteins that accelerates the growth of cancer and develop drug resistance in patients. To address these limitations of cytotoxic chemotherapy, researchers are keenly interested in natural products as some recent studies proved their chemo-protective properties such as anticancer properties [15]. Natural therapies, such as the use of plant-derived products in can-cer treatment, may reduce adverse side effects. Currently, a few plant products are being used to treat cancer. The list of phytochemicals is collected from the literatures [24, 25]. There are different group of phytochemical available from dif-ferent natural products. In this paper, only 21 phenolics are considered for the study. The list of phenolics are given in Table 1. We then searched these 21 phe-nolics in the PCIDB database [26]. For each of the phenolic, the interacting genes are collected. Moreover, the numbers that a phenolic interacting with a gene are also downloaded in the same way. From the lit-eratures [22, 24, 27], satisfactory clinical instances are achieved for Allium sativum, camptothecin, curcumin, green tea, Panax ginseng, resveratrol, Rhus verniciflua and Viscum album dence to support their anticancer effects. The experiments on natural products clearly show that they can be used as complementary therapeutics against various types of cancer.

**Table 1:** List of phytochemical compounds from phenolic group

Sl #	Phenolic	Chemical formula	Sources
1	Curcumin	*C*21*H*20*O*6	Turmeric
2	Gossypol	*C*30*H*30*O*8	Cotton Plant
3	6-Shogaol	*C*17*H*24*O*3	Ginger
4	6-Gingerol	*C*17*H*26*O*4	Ginger
5	Apigenin	*C*15*H*10*O*5	parsley, celery, rosemary, coriander, cloves, spinach
6	Baicalein	*C*15*H*10*O*5	Scutellaria Baicalein, Scutellaria lateriflora
7	Cyanidin	*C*15*H*11*O*6	cranberries
8	Delphinidin	*C*15*H*11*O*7	Grapes, Cran berries, Corn cord grapes, Pomegranates
9	Embelin	*C*17*H*26*O*4	Japanese Ardisia herb
10	Epigallocatechin	*C*22*H*18*O*11	Green Tea
11	Fisetin	*C*15*H*10*O*6	strawberry, apple, grapes, onion, cucumber
12	Genistein	*C*15*H*10*O*5	Fara beans, Soybeans, Psoralea Flemingia vestita, F.macrophylla, Coffe
13	Glabridin	*C*20*H*20*O*4	licorice(root extract)
14	Isoliquiritigenin	*C*15*H*12*O*4	Root of licorice (Glycyrrhiza uralensis)
15	Luteolin	*C*15*H*10*O*6	Cabbage, spinach, peppers
16	Pterostilbene	*C*16*H*16*O*3	Blueberries, almond, mulberries
17	Quercetin	*C*15*H*10*O*7	Fruits, Vegetables, Leaves and Grains
18	Resveratrol	*C*14*H*12*O*3	red and purple grapes, blueberries, cranberries, mulberries, peanuts, roots of Japanese knotweed
19	Rosmarinic	*C*18*H*16*O*8	rosemary, lemon balm, sage, basil
20	Silibinin	*C*25*H*22*O*10	Extract of Milk thistle seeds
21	Psoralidin	*C*20*H*16*O*5	Seeds of Psoralea corylifolia

**Table 2:** Molecular properties of the top five phenolics

Phenolic	Mol. weight	ALogP	#Rotatable Bonds	Polar surface area	HBA (lipinski)	HBD (lipinski)	#Ro5 violations (lipinski)	Aromatic rings
Resveratrol	228.25	2.97	2	60.69	3	3	0	2
Curcumin	368.39	3.85	7	96.22	6	3	0	2
Quercetin	302.24	1.99	1	131.36	7	5	0	3
Epigallocatechin gallate	458.38	2.23	3	197.37	11	8	2	3
Genistein	270.24	2.58	1	90.9	5	3	0	3

**Table 3:** ADME profile for the top five phenolics

Phenolic	Water solubility log(mol/L)	Caco-2 permeability 10^6^ cm/s	Intestinal Absorption (human) % absorbed	Skin permeability	BBB permeability	CNS permeability	Max. dose (human) log mg/day	Hepato-toxicity	Skin sensation	T. Pyri-formis toxicity log ug/L	Minnow toxicity log mM
				Area logKp	logBB	logPS					
Resveratrol	− 3.192	1.191	89.057	− 3.16	− 0.041	− 2.098	0.486	No	No	1.072	0.342
Curcumin	− 4.208	0.55	85.652	− 2.744	− 0.992	− 2.959	− 0.219	No	No	0.372	− 0.631
Quercetin	− 3.17	0.162	74.535	− 2.735	− 1.461	− 3.374	0.983	No	No	0.317	0.943
Epigallocate-chin gallate	− 2.893	− 0.721	58.337	− 2.735	− 2.363	− 4.407	0.473	No	No	0.285	3.239
Genistein	− 3.415	1.019	93.39	− 2.737	− 0.979	− 2.156	0.709	No	No	0.528	5.12

**Table 4:** Phenolics-diseases association network analysis

Phenolic	Disease	# genes inference network	# CSC genes inference network	Inference score	# Ref
Resveratrol	Prostatic	291	131	328.99	252
	Neoplasms				
	Carcinoma	270	116	324.69	125
	Hepatocellular				
	Breast	280	171	273.85	305
	Neoplasms				
	Neoplasms	134	86	145.65	115
	Metastasis				
	Colorectal	125	74	140.51	103
	Neoplasms				
Curcumin	Breast	153	122	247.14	208
	Neoplasms				
	Prostatic	136	94	217.95	192
	Neoplasms				
	Carcinoma	117	77	186.10	84
	Hepatocellular				
	Stomach	83	63	147.58	69
	Neoplasms				
	Neoplasms	81	66	146.98	78
	Metastasis				
Quercetin	Carcinoma	265	111	304.07	120
	Hepatocellular				
	Prostatic	274	135	283.06	233
	Peoplasms				
	Breast	260	154	236.81	289
	Neoplasms				
	Stomach	144	81	153.43	86
	Neoplasms				
	Colorectal	124	70	134.19	108
	Neoplasms				
Epigallocatechin	Prostatic	142	75	133.23	160
Gallate	Neoplasms				
	Carcinoma	119	61	108.13	84
	Hepatocellular				
	Breast	128	87	96.20	189
	Neoplasms				
	Stomach	75	49	73.46	59
	Neoplasms				
	Colorectal	64	43	63.29	67
	Neoplasms				
Genistein	Prostatic	236	123	285.98	236
	Neoplasms				
	Breast	206	167	199.41	232
	Neoplasms				
	Carcinoma	202	98	241.78	106
	Hepatocellular				
	Stomach	122	76	149.43	69
	Neoplasms				
	Lung	111	84	98.88	133
	Neoplasms

**Table 5:** Biological relevance of the resultant phenolics

Phenolic	Top 10 interacting genes	Top 5 pathways (*p*-value)	Top 5 GO terms (*p*-value)
Resveratrol	TNF, SIRT1, IL1B, CASP3, PTGS2, IL6, TP53, RELA, MAPK1, MAPK3	Metabolism–REACT:R-HSA-1430728(4.90e–324), immune system–REACT:R-HSA-168256 (7.89e–284), signal transduction–react:R-HSA-162582 (7.46e–256), innate immune system–REACT:R-HSA-168249 (4.62e–202), metabolic pathways–KEGG:hsa01100 (1.20e–176)	BP: positive regulation of cytosolic calcium ion concentration (2.82e–34), BP: glucose import (9.96e–25), BP: cytosolic calcium ion transport (1.94e–24), BP: internal peptidyl-lysine acetylation (1.00e–17), MF: E-box binding (3.34e–15)
Curcumin	TNF, HMOX1, NFE2L2, BCL2, RELA, CASP3, PTGS2, IL1B, IL6, BAX	Immune system–REACT:R-HSA-168256 (3.15e–178), innate immune system–REACT:R-HSA-168249 (3.11e–143), signal transduction–REACT:R-HSA-162582 (2.18e–142), pathways in cancer-KEGG:hsa05200 (2.08e–141), cytokine signaling in immune system–REACT:R-HSA-1280215 (1.24e–122)	BP: positive regulation of cytosolic calcium ion concentration (1.78e–20), BP: cytosolic calcium ion transport (3.15e–14), BP: glucose import (1.14e–13), BP: activation of protein kinase B activity (1.66e–12), BP: T-helper cell differentiation (8.13e–12)
Quercetin	TNF, CASP3, HMOX1, IL1B, NOS2, MAPK3, NFE2L2, MAPK1, BCL2, AKT1	Immune system–REACT:R-HSA-168256 (6.77e–271), signal transduction–REACT:R-HSA-162582 (1.81e–247), metabolic pathways–KEGG:hsa01100 (9.34e–193), innate immune system–REACT:R-HSA-168249 (1.30e–178), pathways in cancer–KEGG:hsa05200 (1.96e–144)	BP: positive regulation of cytosolic calcium ion concentration (1.63e–23), BP: glucose import (7.99e–22), BP: cytosolic calcium ion transport (4.09e–16), BP: protein polyubiquitination (1.81e–14), BP: internal peptidyl-lysine acetylation (1.80e–12)
Epigallocatechin gallate	ARNTL, AKT1, BDNF, CAT, CREB1, SOD1, BNIP3, CLOCK, COX2, COX4	Tuberculosis–KEGG:hsa05152 (1.03e–21), Hepatitis B–KEGG:hsa05161 (5.22e–21), Non-alcoholic fatty liver disease (NAFLD)–KEGG:hsa04932 (8.26e–21), AGE-RAGE signaling pathway in diabetic complications–KEGG:hsa04933 (6.25e–19), Toxoplasmosis–KEGG:hsa05145 (2.85e–18)	MF: E-box binding (6.31e–9), BP: internal peptidyl-lysine acetylation (1.95e–6), MF: RNA polymerase II proximal promoter sequence-specific DNA binding (4.55e–10), BP: positive regulation of protein serine/threonine kinase activity (4.97e–10), BP: activation of protein kinase activity (6.94e–9)
Genistein	ESR1, ESR2, CFTR, CASP3, TNF, PGR, CYP1A1, MAPK3, MAPK1, PTGS	Immune System–REACT:R-HSA-168256 (2.97e–210), Metabolism–REACT:R-HSA-1430728 (6.69e–207), Signal Transduction–REACT:R-HSA-162582 (9.81e–201), Innate Immune System-REACT:R-HSA-168249 (1.01e–158), Pathways in cancer–KEGG:hsa05200 (3.10e–137)	BP: positive regulation of cytosolic calcium ion concentration (4.38e–31), BP: cytosolic calcium ion transport (3.97e–19), BP: glucose import (2.80e–14), BP: iron ion homeostasis (3.87e–12), BP: T-helper cell differentiation (4.24e–12)

### DisGeNet

DisGeNet is a database that yields scores to the genes depending on various metrics [28]. Here, the DSI and DPI scores for each gene are considered. The DSI score of a gene indicates how much a gene is specific to a disease. For example, if a gene is associated with too many diseases, DSI score for that gene is as low as 0. On the other hand, if a gene is associated with only one or few diseases, its DSI score would be as high as 1. It is calculated as Eq. 1:

1 $$DSI = \frac{{log_{{2}} \left( {N_{d} /N_{T} } \right)}}{{log_{{2}} \left( {{1}/N_{T} } \right)}},$$

where *N*_*d*_ is the number of diseases associated to the gene and *N*_*T*_ is the total number of diseases in DisGeNet. The DPI score for a gene is 1 if it is associated with largely different classes of diseases and 0 if it is associated with same class of diseases. It is calculated according to Eq. 2.

2 $$DPI = \, \left( {N_{dc} /N_{T \, C} } \right) * {1}00,$$

where *N*_*dc*_ is the number of the different MeSH disease classes of the diseases as-sociated to the gene and *N*_*T C*_ is the total number of MeSH diseases classes in DisGeNet.

### PageRank (PR)

PR invented by Google founders Larry Page and Sergey Brin, is a way of measuring the importance of website pages [29]. PR is an algorithm used by Google Search to rank websites in their search engine results. Essentially, PR does not rank web sites as a whole, but is determined for each page individually. Further, the PR of page A is recursively defined by the PR of those pages which link to page A. When site A links to any web page, Google considers this as site A endorsing, or casting a vote for that page. Google takes into consideration all of these link votes (i.e., the website’s link profile) to draw conclusions about the relevance and significance of individual webpages and your website as a whole. This is the basic concept behind PR. In short, PR is”vote” by all the other pages on the Web regarding how important a page is. A link to a page counts as a vote of support. When there is no link, it means no support (but it is an abstention from voting rather than a vote against the page). From the original Google paper [29], PR has been defined as in Eq. 3.

3 $$PR\left( A \right) = \left( {{1} - \, d} \right) + d\left( {P \, R\left( {T{1}} \right)/C\left( {T{1}} \right) + ... + PR\left( {T \, n} \right)/C\left( {T \, n} \right)} \right),$$

where PR(A) is the PR of page A, PR(*T*_*i*_) is the PR of pages *T*_*i*_ which links to page A, C(*T*_*i*_) is the number of outgoing links on page Ti as each page spreads its vote out evenly amongst all outgoing links. The number of outgoing links for page 1 is C(*T*_1_), C(*T*_*n*_) for page n, and so on for all pages, and d is a damping factor which can be set between 0 and 1. They usually set d to 0.85. Note that the PR form a probability distribution over pages, so the sum of all web pages’ PR will be 1. PR or PR(A) can be calculated using a simple iterative algorithm, which corresponds to the principal eigenvector of the normalized link matrix of the web.

PR or PR(A) can be calculated using a simple iterative algorithm, and corresponds to the principal eigenvector of the normalized link matrix of the web. That means just calculating a page’s PR without knowing the final value of the PR of the other pages. Basically, in each run, the calculation is getting closer to estimate the final value. So, repeat the calculations many times until the numbers stop changing by a threshold value.

### Preprocessing of the dataset

Assume that *p* = *{p*_1_*, p*_2_*, …., p*_*m*_*}* is a list of the phenolics whose set of interacting genes are *G*_*p*_ = *{G*_*p*1_*, G*_*p*2_*, …., G*_*pm*_*}*, where *G*_*p*1_, *G*_*p*2_,… *G*_*pm*_ is the gene set that interacts with *p*_1_, *p*_2_, …., *p*_*m*_ respectively. CSC genes are collected from CSCdb database. Suppose *q* CSC genes are collected and described as *CSC* = *{cg*_1_*, cg*_2_*, …., cg*_*q*_*}*. We then take the common genes between each interacting set and CSC gene set and it generates *G*_*p*1_ ∩ *CSC, G*_*p*2_ ∩ *CSC, ….., G*_*pm*_* ∩ CSC* and it implies *G*_1_*, G*_2_*, …, G*_*m*_ if *G*_*p*1_ ∩ *CSC* = *G*_1_, *G*_*p*2_ ∩ *CSC* = *G*_2_…, etc. Next, out of these gene sets, the common gene set *s* is taken out and then these genes are searched in DisGeNet database for collecting their score. A few of them do not have scores; therefore, they are excluded from the set. Finally, *n* genes (< *s*) are gathered for further processing.

### Proposed method

**Fig. 1:**
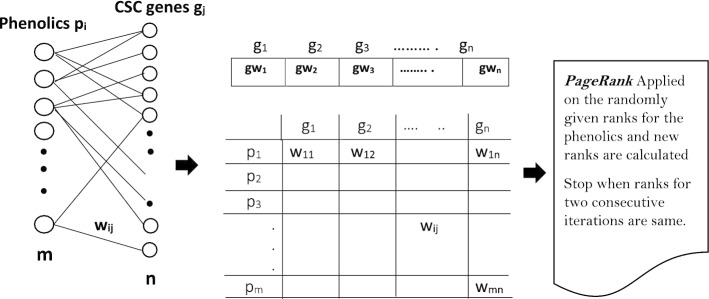
Pipeline of the proposed method

From the collected datasets, a weighted bipartite graph is constructed where one set of the bipartite graph is the set of phenolics (i.e., *p*) and other set is the gene set (i.e., *n*). The edges are weighted according to the number of ways a phenolic interacting with the genes. These weights are normalized by using mean and standard deviation. The absolute of the normalized values are taken into consideration. The *n* genes are also weighted in terms of DSI and DPI scores. Given the above weighted bipartite graph, the job of the algorithm is to rank the phenolics. Here, it comes the concept of Page Ranking that has been used to build our model. Starting with a random ranking for the phenolics, the edge weights and gene weights are used to recalculate the new ranks and gradually conclude the final ranks for the phenolics. The critical question is when to stop recalculating the ranks for the phenolics. The answer is kept on calculating the ranks for the phenolics until no change is found in the last two rankings. The pictorial definition of the proposed method has been shown in Fig. 1.

Rank calculation: Let *p*_1_ is the phenolic for which a random rank *r*_1_ is given initially. A random value between 0 and 1 has been generated for each pheno-lic. When these values are sorted in non-increasing order, they will produce the rankings for the phenolics. So, *r*_1_ is the value in between 0 and 1. If phenolic *p*_1_ interacts with *x* genes with edge weights *w*_1_*, w*_2_*, …, w*_*x*_ and *x* genes have the weights *gw*_1_*, gw*_2_*, …, gw*_*x*_ given by DSI and DPI, then the new rank of phenolic *p*_1_ is calcu-

$${\text{lated}}\;{\text{as}}\;r_{new} = r_{{1}} \sum_{i = 1} w_{i} * gw_{i} /abs\left( {normalized\left( x \right)} \right)$$

## Results and discussion

Among 21 phenolics, the top five phenolics are *Resveratrol*, *Curcumin*, *Quercetin*, *Epigallocatechin Gallate* and *Genistein*. For demonstration purpose, only these top ranked phenolics are studied for their oral bioavailability through molecular properties, drug likeness through pharmacokinetic properties and associated net-work with CSC genes.

### Calculation of molecular properties

All the calculated parameters, namely molecular weight, log P, the number of rotat-able bonds, polar surface area, the number of hydrogen bond donors and acceptors, the Lipsinki Rule violation, aromatic rings and heavy atoms, are thought to be as-sociated with molecular flexibility, oral bioavailability, solubility and permeability of drugs which are the basic requirements for any drug to have good pharmacoki-netic parameters. These properties are calculated from ChEMBL, a large bioactiv-ity database [30]. The molecular weight describes the molecular flexibility and oral bioavailability. As summarized in Table 2, the molecular weights for all the five phe-nolics are 228*.*25, 368*.*39, 302*.*24, 458*.*38 and 270*.*24, respectively. This information indicates that the top ranked phenolics have high molecular flexibility as well as oral bioavailability. It has been seen that the molecular flexibility correlates with molecular weight, that is, larger compounds would be more flexible. The *logP* is lipophilicity of a compound and for all the five phenolics, *logP* values are greater than or equal to 2, but less than 5. The numbers of rotatable bond are defined as any single bond, not in a ring, bound to a nonterminal heavy atom(i.e., non-hydrogen). It can be seen the majority of compounds with seven or fewer rotatable bonds met which represents more oral bioavailability as published in the literature [31]. As Polar Surface Area (PSA) characterizes drug absorption, including intestinal ab-sorption and bioavailability, therefore the five phenolics have high PSA, specially *Epigallocatechin Gallate* (197*.*37) as PSA. From literature [31], it has been estab-lished that 12 or fewer Hydrogen Bond (H-Bond) Acceptors (HBA) and H-Bond Donors (HBD) are essentially good for those with high oral bioavailability. In this study we found top ranked phenolics have less than 12 HBAs and HBDs. Lipinski rule of 5 based on five criteria namely, molecular mass, high lipophilicity (*logP*), hydrogen bond donors, hydrogen bond acceptors and molar refractivity. Except for *EpigallocatechinGallate*, no top ranked phenolics are violated the Lipsinki rule of 5. It has been well established that more than three aromatic rings in a molecule correlate with poorer drug development ability [32]. All the top five phenolics have 3 or fewer aromatic rings, indicating their draggability.

### Phytochemical and structural properties

#### Resveratrol

**Fig. 2:**
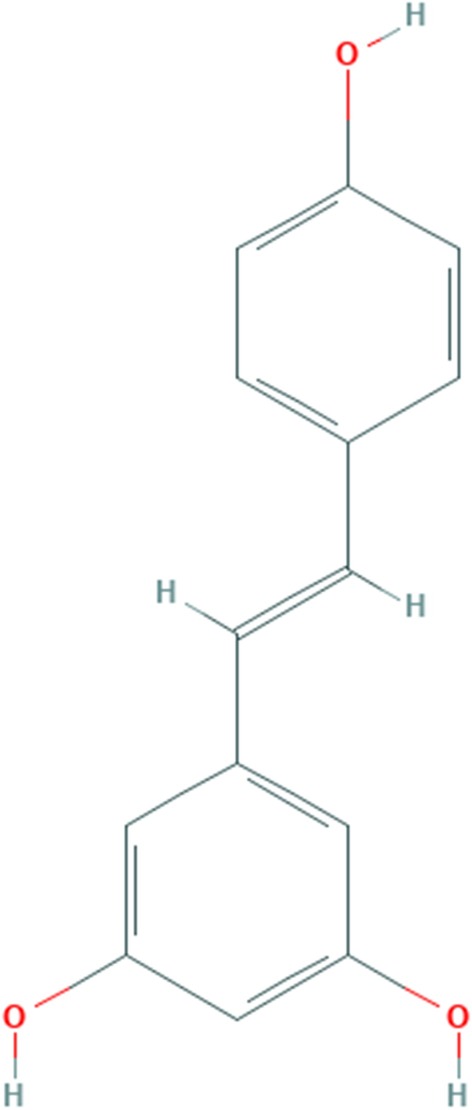
Chemical bonding of resveratrol

The phytochemical compound is stilbenoids. A stilbenol is stilbene in which the phenyl groups are substituted at positions 3, 5, and 4′ by hydroxy groups. The chemical structure of resveratrol is given in Fig. 2. It has anticancer properties and inhibits lipid peroxidation of low-density lipoprotein and prevents the cytotoxicity of oxidized LD [33]. Resveratrol also increases the activity of some antiretroviral drugs in vitro.

### Curcumin

**Fig. 3:**
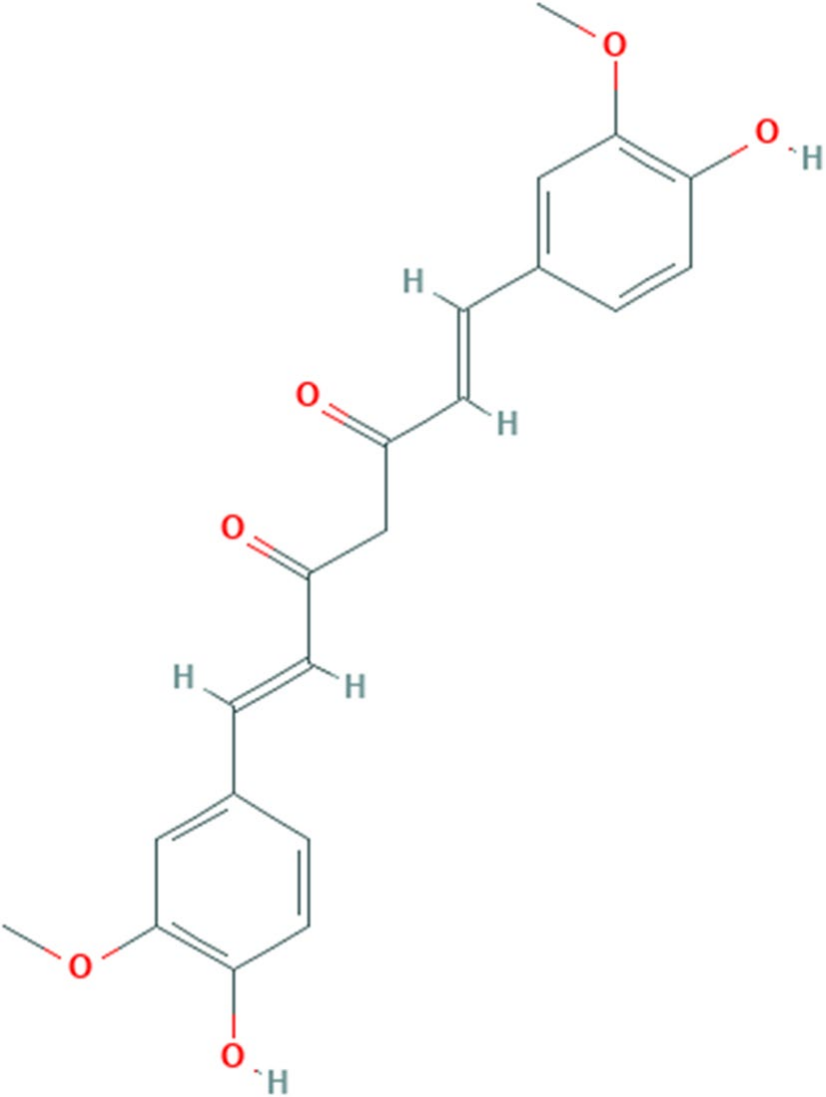
Chemical bonding of curcumin

The phytochemical compound is Diarylheptanoids. A beta-diketone is methane in which two of the hydrogens are substituted by feruloyl groups. A natural dyestuff is found in the root of Curcuma longa. Curcumin has antioxidant, anti-inflammatory, antiviral and antifungal actions [34, 35]. The chemical structure of curcumin is given in Fig. 3.

### Quercetin

**Fig. 4:**
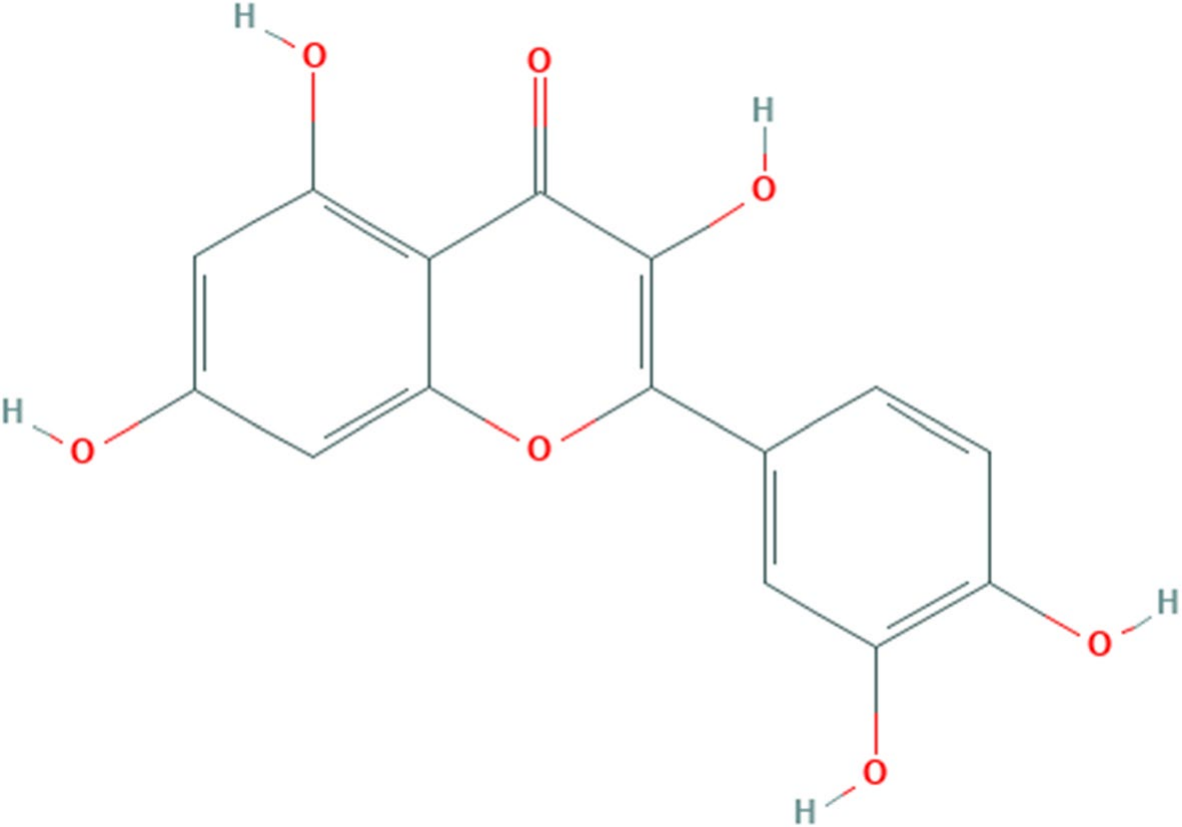
Chemical bonding of quercetin

The phytochemical compound is flavonoid. A pentahydroxyflavone has the five hy-droxy groups placed at the 3-, 3′-, 4′-, 5- and 7-positions. It is one of the most abundant flavonoids in edible vegetables, fruit and wine. Health effects include an improvement of cardiovascular health, reducing risk for cancer, and protection against osteoporosis. This phytochemical has anti-inflammatory, anti-allergic and antitoxic effects [36]. The chemical structure of quercetin is shown in Fig. 4.

### Epigallocatechin gallate

**Fig. 5:**
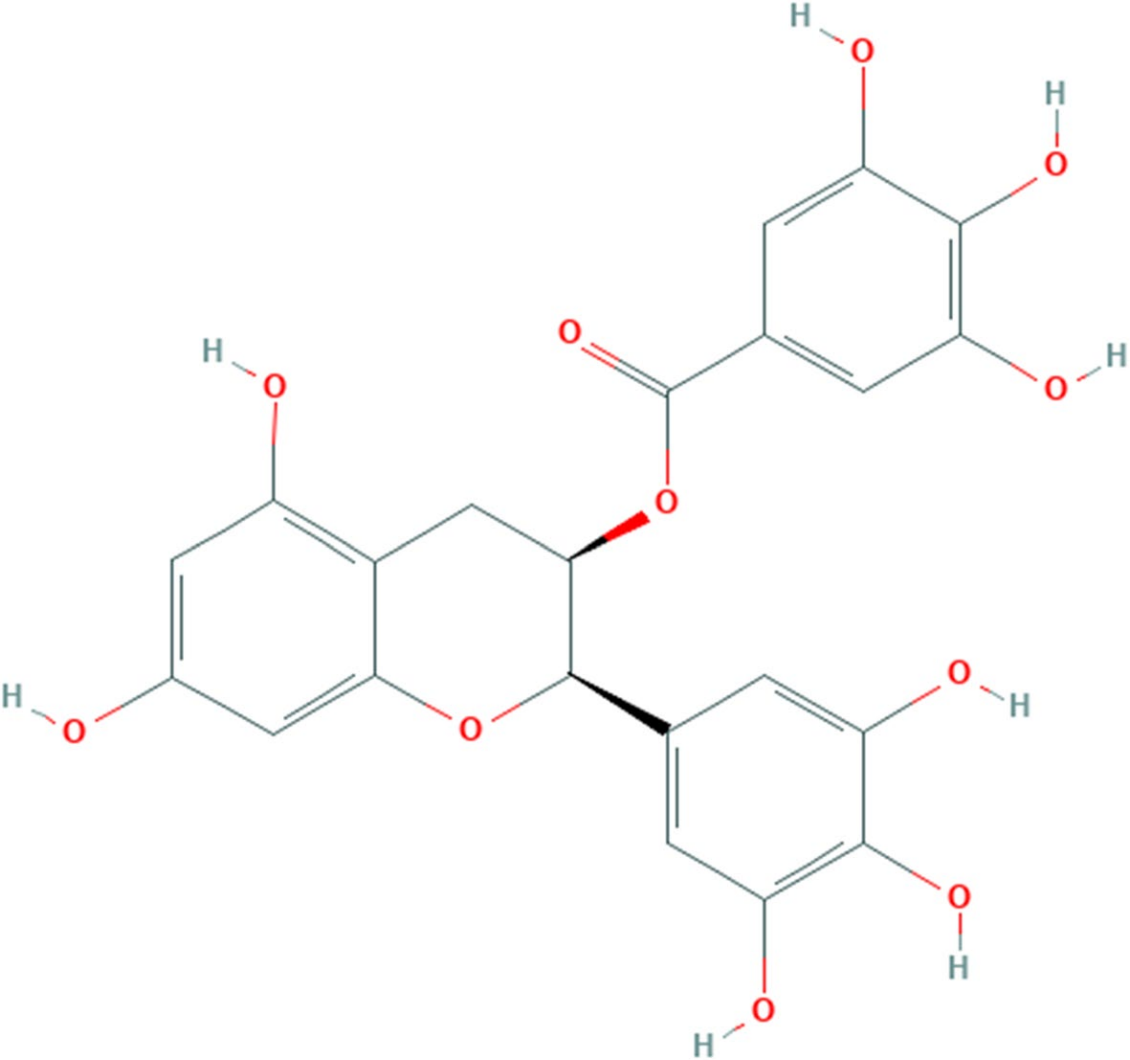
Chemical bonding of Epi-Gall

The phytochemical compound is Flavan 3-ols flavan. A gallate ester obtained by the formal condensation of gallic acid with the (3R)-hydroxy group of (-)-epigallocatechin. A number of chronic diseases have been associated with free rad-ical damage, including cancer, arteriosclerosis, heart diseases and accelerated ag-ing [37]. Epigallocatechin gallate interferes with many enzyme systems: it inhibits fast-binding and reversible fatty acid synthase, increases tyrosine phosphorylation of the insulin receptor, activation of ornithine decarboxylase. The chemical structure of epigallocatechin gallate is given in Fig. 5.

### Geninstein

**Fig. 6:**
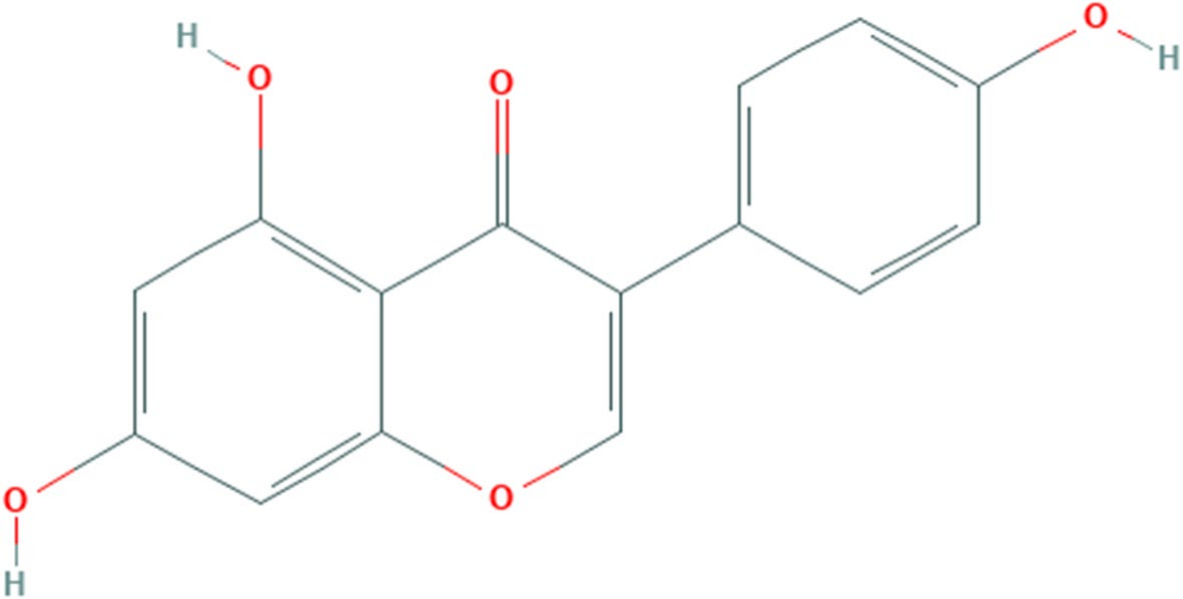
Chemical bonding of Genistein

The phytochemical compound is Isoflavones, 7-Hydroxyisoflavone with additional hydroxy groups at positions 5 and 4′. It is a phytoestrogenic isoflavone with antiox-idant properties. it acts as a phytoestrogens, antioxidant, anti-cancer agent and it could help people with metabolic syndrome [38]. The chemical structure of gninstein is given in Fig. 6.

### Drug likeliness analysis

The pharmacokinetic properties of a chemical present the drug-like ability of a molecule. Therefore, it is an important aspect in consideration. These pharma-cokinetic properties are calculated in pkCSM platform [39]. Water Solubility of a compound (logS) reflects the solubility of the molecule in water at 25^*◦*^*C* and given as the logarithm of the molar concentration *logmol/L*. A compound is considered to have high Caco-2 permeability if it has a *P app* > 8 ∗ 10^*−*6^ cm/s. High Caco-2 permeability would be for a predicted value > 0*.*90. From Table 3, it is clear that most of them are greater than 0*.*90 as Caco-2 permeability. For a given compound, the intestinal absorption predicts the percentage that will be absorbed through the human intestine. A molecule with an absorbance of less than 30% is considered to be poorly absorbed. All the top five ranked phenolics from our experiment have intestinal absorption values greater than 30%. A compound is considered to have a relatively low skin permeability if it has *logKp* > *− *2*.*5. The outcome of our experiment shows that all the top five ranked phenolics have *logKp* > *− *2*.*5. The ability of a drug to cross the brain is an important measure to reduce the side effects. Blood–Brain permeability is measured as the logarithmic ratio of the brain to plasma drug concentration (*logBB*). For a given compound, a *logBB* > 0*.*3 has been treated as readily cross the blood–brain-barrier(BBB) while molecules with *logBB* < *− *1 are poorly distributed to the brain. The results from the table indi-cates that except Quercetin and Epigallocatechin Gallate, the rest three phenolics have good BBB values which are − 0*.*041, − 0*.*992 and − 0*.*979. They have same for the Central Nervous System (CNS) permeability. The maximum recommended tol-erated dose provides an estimate of a toxic dose threshold of chemicals in humans. Hepatoxide predicts whether a given compound is likely to be associated with dis-rupted normal function of the liver. Skin sensation indicates whether the compound is skin sensitive. All the top five phenolics are neither hepatoxic nor skin sensitive. Another toxicity measure is T. Pyriformis value, which is considered toxic if the predicted value is greater than − 0*.*5*logug/L*. The T.Pyriformis values of all the top phenolics are (1*.*072, 0*.*372, 0*.*317, 0*.*285 and 0*.*528) greater than − 0*.*5*logug/L*. The predicted Minnow toxicity value is regarded as high acute toxicity if it is below 0*.*5* mM* (*logLC*50 < *− *0*.*3). It is evident that the top five phenolics are not Minnow toxic (Table 3).

### Association with CSC genes

To find the association between the top ranked phenolics and CSC genes, the Com-parative Toxicogenomics Database (CTD) [40] has been used. As shown in Table 4, a majority of them are associated with prostatic neoplasms, breast neoplasms, car-cinoma hepatocellular, stomach neoplasms, and colorectal neoplasms, as sorted by their inference score. The table also shows the association between phenolics and diseases (neoplasms class) by interacting with the cancer genes in the inference network and CSC genes that has also been tabularized. Then the inference score of the network and references are collected from the CTD database. It has been noticed that all the phenolics interact with the highest number of CSC genes of Breast neoplasms. Biological relevance of the top rank phenolics are also described in Table 5. To find biological relevance computationally, top ten interacting genes, top five pathways with *p*-value and top five GO terms with *p*-value are collected from CTD database. It is clear from the table that most of the top interacting genes are cancer related, however it is still unknown whether they are also CSCs related. However, there are many works conducted regarding the combinations of the drugs targeting different CSC-genes [41–44].

The total experiment has been done computationally. From dataset collection to validating the top ranked phenolics, our results relied on the information from different databases and literatures. However, we will extend the study not only on CSC related genes but their draggability in future.

## Conclusions

The phenolics have already been reported to have significant anti-cancer potential. Here, we further explored them for their mechanistic perspective as potential anti-cancer lead molecules for CSC genes. Computationally, a bipartite graph has been formed where one group is the set of collected CSC genes and the other group is the interacting phenolics. The edges represent the interactions and are weighted accord-ing to the strength of interaction between the phenolics and the CSC genes. Also, the CSC genes are given some weight by two metrics, namely, *DSI* and *DP I*. Then, a ranking technique inspired from PR algorithm has been developed to rank the phe-nolics. However, one can apply other ranking algorithms (e.g., matrix factorization) to rank the phenolics. The ranks of the phenolics indicate their association with the CSC genes. From data collection to validation, several databases have been used. In this study, few phytochemicals have been tested and validated for their strong effects on CSCs. Further efforts should be made to experimentally validate their potential to target CSCs, toxicities and drug-abilities. The associated pathways for all the top ranked phenolics are related to cancer, immune system, metabolic, signal transduction etc. Moreover, the low *p*-values associated with the pathways indicate the statistical significance of the phenolics to those pathways. Lower p-values of the GO-terms indicate that the resultant phenolics are statistically significant and are not selected randomly and it is evident from the table. As future work, we will extend our work through including the combinations of the drugs targeting differ-ent CSC-genes into our current study, as well as collecting more data for a larger number of phenolics.

## Data Availability

The following publicly available databases have been used in this study namely CSCdb database (https://bioinformatics.ustc.edu.cn/cscdb), PCIDB database (https://genome.jp/db/pcidb), DisGeNET database (https://disgenet.org/), ChEMBL database (https://ebi.ac.uk/chembl/), pkCSM database (https://biosig.unimelb.edu.au/pkcsm/) and CTD (https://ctdbase.org/).

## Abbreviations

CSCs: Cancer stem cells
DSI: Disease specificity index
DPI: Disease pleiotropy index
PR: PageRank
PSA: Polar surface area
HBA: Hydrogen bond (H-Bond) acceptors
HBD: Hydrogen bond (H-Bond) donors
BBB: Blood–brain-barrier
